# Supplementary material for: High-Temperature-Responsive Poplar lncRNAs Modulate Target Gene Expression via RNA Interference and Act as RNA Scaffolds to Enhance Heat Tolerance
Source: Int J Mol Sci. 2020 Sep 16;21(18):6808. doi: 10.3390/ijms21186808 (PMC7555564; doi:10.3390/ijms21186808)
Supplement: Supplementary file 1 [file ijms-21-06808-s001.zip › Supplemental Figure S1-S12.doc]

**Figure S1.** Antioxidant enzyme system time course analysis under heat stress. Data are mean ± SE of three separate measurements. Error bars represent standard error. The significant differences of biochemical characteristics of poplar in response to time course of heat stress were determined through Fisher’s Least Significant Difference (LSD) test. Different letters on error bars indicate significant differences at *P* < 0.01 among different time points.

**Figure S2.** Transcript abundance of high temperature-responsive ncRNAs. Accumulate frequency of transcript abundance of high temperature-responsive lcncRNAs and mRNA.

**Figure S3.** Scatterplots of lncRNAs and mRNAs. Red spots represent lncRNAs; blue spots represent mRNAs.

**Figure S4.** GO enrichment of target genes that co-expressed with high temperature-responsive lncRNAs. Green histogram represents enrichment of target genes that co-expressed with high temperature-responsive lncRNAs.

**Figure S5.** Location of TCONS_00202587 and its targets. Yellow box represent the location of TCONS_00202587 in genome. Balck arrows represent the direction of TCONS_00202587 transcription. Skyblue box represent the location of lncRNA neighboring genes. Red arrows represent the direction of neighboring genes transcription.

**Figure S6.** Details of secondary structure and sequences of TCONS_00202587. IR1 and IR2 represent two potential interaction region which binding with promoter of targets. Schematic diagram of secondary structure mutants. TCONS_00202587^-∆stem-loop3^ and TCONS_00202587^-∆stem-loop4^ significantly changes its secondary structure.

**Figure S7.** Temporal expression patterns of lncRNA and their targets under high temperature treatment. A. Temporal expression patterns of TCONS_00260893 and Potri.017G0089800 different isoforms under high temperature treatment. B. Temporal expression patterns of TCONS_00202587 and Potri.012G002700 different isoforms under high temperature treatment. Relative transcript levels were calculated by real-time PCR with Actin as the standard. Data are mean ± SE of three separate measurements. Error bars represent standard error.

**Figure S8.** Location of TCONS_00260893 and different transcripts of targets**.** A:the location of TCONS_00260893 and seven transcripts of Potri.017G089800 in genome. Skyblue box represent the location of TCONS_00260893 in genome. B: The location of seven transcripts of Potri.017G089800 and heat responsive siRNA cluster in genome.

**Figure S9.** Schematic diagram of lncRNAi and lncRNAe. For mimicking silencing and overexpression of lncRNAs, antisense RNA and sense RNA strands, respectively, were synthesized with Fluorescein 5-isothiocyanate (FITC) at the 5’ end. The LDH-lactate-NS colloid in Murashige and Skoog (MS) medium was added drop-wise to lncRNAs at a volume ratio of 3:1 followed by gentle mixing. The mixture was incubated for 1 h to form the LDH-lactate-NS–lncRNA conjugate. Poplar roots were dipped in liquid MS medium with 0.2 μg/μL LDH-lactate-NS– lncRNA conjugate. After incubation at room temperature for 3 h, the roots were rinsed several times with a standard growth medium and then observed under an Olympus BX61 microscope for fluorescence imaging.

**Figure S****10.** Tissue specific expression pattern of two candidate lncRNAs and their targets. A: Tissue specific expression pattern of TCONS_00260893 and Potri.017G089800 six isoforms. B: Tissue specific expression pattern of TCONS_00202587 and Potri.012G002800. Letters with different color on error bars indicate significant differences at *P* < 0.01. The significant differences of each genes among young leafs, phloem, cambium root and apex were determined through Fisher’s Least Significant Difference (LSD) test. Letters with different colors on error bars indicate significant differences of gene with the same color among different groups at *P* < 0.01.

**Figure S11.** The survival rate of wild-type, Potri.012G002800-OE and Potri.017G089800-OE trangenic plant under heat stress. Letters with different color on error bars indicate significant differences at *P* < 0.01.

**Figure S12.** Correlation of qPCR and RNA-seq data.
